# Health Tract, No. 85

**Published:** 1865

**Authors:** 


### HEALTH TRACT, No. 85.

### THE SABBATH REST.

No one muscle of the body, no one set of muscles can be continuously used, without an eventual paralysis, or total loss of power, until restored by rest. But if one class of muscles be employed for a time, then another, while the former is at rest, the two thus alternating may be kept in motion, without the slightest fatigue, for hours together. A child may even cry with the weariness from walking; but present him suddenly with a beautiful little wagon, and allow him to take hold of it and draw a companion over a smooth road, the offer will be accepted with alacrity, and the amusement will continue for a time equal to the walk, without any complaint of being tired; on the contrary, there will be a freshness of action, new and delightful. Many a traveler has rested himself from riding on horseback or in a carriage, by alighting and walking a mile or more; simply because a different combination of muscular action is brought into play; either a new set of muscles, or an action of the old ones in a different direction; all going to show that the muscular system, the whole body, will have rest, or must prematurely perish. Precisely alike is the law of the mind, whose faculties are various. A man who thinks intently upon a single subject becomes incapable at length of concentrating his thoughts upon that subject to advantage, and instinctively lays down his book, his model, or his pen, to take a walk. It is an observed fact, that a large number of professed students of prophecy become deranged; the world is full of monomaniacs, of persons who have so persistently thought of a single subject, that the mind has become permanently "unhinged" in regard to it. The attention of the French government has lately been drawn to the alarming fact, that "one in every ten of the scientific branches of the army finishes his course in a lunatic asylum, in consequence of the severe attention to mathematical training." The rector of the training-college of Glasgow says, from long and extensive observation, he "will undertake to teach a hundred children in three hours a day as much as they can possibly receive;" that is, when a child has been kept at study three hours, its brain becomes incapable of pursuing it further advantageously, until rested. These things show that unless mind and body both have rest, both will be destroyed; and to save both, Divine wisdom issued the precept, "in the beginning," "On the seventh day thou shalt rest." It was no arbitrary command; it was an injunction fraught with wisdom and benevolence; and in this sense was it that "the Sabbath was made for man;" made to save his body from premature wearing out, and his mind from fatuity, by diverting it for one seventh of the time from its ordinary studies and affections, and fixing it on a totally different class; taking it away from the wasting, wearing harassments and jarrings and anxieties of business, to employ it in the contemplation and worship of Divinity, to soothe, to elevate and sanctify; compelling us to exclaim in affectionate admiration, not only as to the laws of our physical, but as to those of our moral nature: "In loving-kindness hast thou made them all!" The observation of the laborer and the business-man will testify to the exhaustion which Saturday night always brings, and to the renewed alacrity with which business is hurried to on Monday mornings. The reflecting know that without the compulsory observance of the Sabbath-day, multitudes of helpless slaves, of defenseless apprentices, of dependent employés; the uncomplaining horse, and ox, and mule; would be driven to death! Who can deny after this, that the Bible Christianity is the poor man's friend? And yet how many malign that blessed book, and wage a relentless and life-long war against that religion!

